# Supplementary material for: Ensemble dynamics and information flow deduction from whole-brain imaging data
Source: PLoS Comput Biol. 2024 Mar 15;20(3):e1011848. doi: 10.1371/journal.pcbi.1011848 (PMC10942262; doi:10.1371/journal.pcbi.1011848)
Supplement: S1 Text — (PDF) [file pcbi.1011848.s001.pdf]

S1 Text - Supporting Files

All files are at the Figshare repository

File 1 Fig. Result of 4D imaging in 24 samples.

Neuronal activities and their cross-correlations are shown color-coded.

File 2 Note. Demonstration of typical temporal dynamics extracted by TDE-RICA.

TDE-RICA was applied to Lorenz attractor as test examples and the results are shown.

File 3 Fig. Results of TDE-RICA for 94 selected neurons of 10 selected samples.

File 4 Fig. Completing missing values by TDE-RICA by matrix factorization.

(Top left) Activity time series of all 177 neurons in the same sample as in Fig 1A. The missing activity of several neurons is shown in white (top left). The missing values are completed in the reconstructed time series by TDE-RICA by matrix factorization (bottom right).

(Top right) Full set of motifs of neural activities obtained by TDE-RICA by matrix factorization.

(Bottom left) Full set of motif occurrences.

(Bottom right) Reconstructed time series. The missing values in the top left panel were well completed.

File 5 Fig. Results of TDE-RICA with matrix factorization for all 177 neurons of all 24 samples.

Neural activity data for all 24 samples obtained by the whole-brain imaging experiments are also included.

Page 1-24: The experimental neural activity data and the results of TDE-RICA with matrix factorization in each sample.

Page 25-48: The phase diagram of all pairs of the motif occurrences in each sample.

Page 49: The motifs obtained by TDE-RICA with matrix factorization.

Page 50: Cross-correlation functions of motif occurrence, averaged across samples.

Page 51-141: Time course and phase diagram of all pairs of the motif occurrences in each sample.

File 6 Note. TDE-RICA to simple time series data with different time-delay steps

TDE-RICA was applied to simple time series including sine, square, and aperiodic square waves with time-delay steps of 10, 30, 60, 100, 150, 300, 600, and 1000.

File 7 Data. TDE-RICA to the whole-brain activity dataset with different time-delay steps

TDE-RICA was applied to the whole-brain activity dataset with time-delay steps of 10, 30, 60, 100, 150, 300, 600, and 1000; in order to reduce memory usage, the data was subsampled by 10 for steps 300, 600, and 1000.

File 8 Fig. Changes in neural activity induced by NaCl stimulation

The trial-by-trial mean and standard deviation of stimulus-triggered neural activity were visualized for each neuron. The absolute value of the difference between the mean value of the 50 post-stimulus frames and the mean value of the 50 pre-stimulus frames was considered the magnitude of the response. For the scatter plots on the first page, the horizontal axis is the magnitude of the response, the vertical axis is the standard deviation of 100 frames before and after stimulation, and the colors represent the neuron type.

File 9 Fig. Cross-correlation functions of motif occurrences averaged across samples.

The red line indicates the mean and the blue lines the mean ± standard deviation. Pairs are highlighted in green if the mean + standard deviation is <0 for any lag or if the mean - standard deviation is > 0 for any lag.

File 10 Fig. Determination of hyperparameters for gKDR-GMM

(A–B) Determination of k' and τ' for gKDR-GMM. (A) gKDR-GMM models were generated for each sample with different embedding parameters k' andτ', and reduction dimension K; the free run simulation was run three times. Simulation results were evaluated by mean square difference between the cross-correlation matrix of neuron activities between real data and simulation results. This figure shows the average of all samples and all repeats (=3 repeats) for all tests. (B) The same results in A shown separately for each sample.

(C–E) Determination of K for gKDR-GMM. (C) gKDR-GMM models were generated for each sample with different reduction dimension K; the free run simulation was run three times. k' =30, τ'=10,

repeat=3 for all tests. In "direct links" only neurons sending direct synaptic input to the target neuron were considered "presynaptic" while in "indirect links," used for missing presynaptic neurons, neurons presynaptic to the missing neuron were also included as "presynaptic neuron." (D) Same results as in (C), but all samples were averaged and SEM are shown. (E) Line plot representation of (D).

(F–H) Determination of hyperparameter for gKDR-GMM. (F) gKDR-GMM models were generated for each sample with different number of Gaussians for GMM as well as reduction dimension *K* for gKDR. "direct links" and "indirect links" are as in (C). (G) Same results as (F) but the average of all samples and all *K* are shown as well as SEM. (H) Line plot representation of (G).

(I–K) Determination of hyperparameter *K* for gKDR-GP. (I) gKDR-GP models were generated for each sample with different reduction dimension K; the free run simulation was run three times. k' =30, τ'=10 for all tests. Only results for "indirect links" are shown. (J) Same results as (I) but the average of all samples and all *K* are shown as well as SEM. (K) Line plot representation of (J).

File 11 Fig. Simulation results of gKDR-GMM

A: $K = 3$, B: $K = 4$, C: $K = 5$.

File 12 Fig. Overview of simulation results of gKDR-GMM

For comparison of real data and simulated data, cross correlation between activities of neuron pairs (as shown in File 11, bottom rows) are plotted for real (x axis) and simulated (y axis) activities for all pairs of neurons in each sample. Results of simulation by different gKDR-GMM models (K=3, 4, 5; kGMM was fixed at 2) are shown.

File 13 Fig. Lagged cross-correlation of all combinations of neurons for all samples

File 14 Fig. Activity profile of example neuron pairs with lagged correlation

File 15 Fig. Simulation results by gKDR-GMM, evaluated by TDE-RICA

File 16 Fig. Periodic mean of neuronal activities to visualize same periodicity as sensory input

File 17 Fig. Probabilistic and deterministic models

A. Simulation result using probabilistic model, deterministic model and deterministic model with random noise

B. Cross correlation of results in A

File 18 Fig. Mean log likelihood values for each neuron in each sample.

A. Neuron activity time-series data were evenly split into three parts (part 1 to 3) and gKDR-GMM models were made by each part (rows, model 1 to 3). The split data for each part (columns) were evaluated by log likelihood determined by the model distribution ($P(y_{i,j}(t+\Delta t)|U_{i,j}(t))$, see methods). Both self- and cross-validation results are shown.

B. Cross validation results.Shown are results of bootstrap test by random permutation of presynaptic data ($X_{i,j}(t)$, see (C) below). p-values of real data (columns) estimated by each permutation results are indicated. Results for all samples and all neurons. Both self- and cross-validation results are shown.

C. Histograms showing the results of bootstrap tests. Blue bars show distribution of mean log likelihood of real data estimated by the model using randomly permutated presynaptic data, $X_{i,j}(t)$. Red triangles show log-likelihood values estimated by using non-permuted data (same as (A)). Histogram distribution was considered normal distribution and p-values were estimated as in (B). Self: model part = test part. Cross x: test part = model part + x (circularly permutated).

File 19 Fig. Estimation of synaptic connection strength.

A. Estimated synaptic weights from different models. Each box shows model-estimated synaptic weights from the neuron on the y axis to the neuron on the x axis. In each box, model-estimated weights are shown in 15 x 3 cells as explained in Fig 7A, where 15 rows are results from models using different *K* for gKDR using offset 0-4 of the data, and 3 columns show results from three parts of split time series. Models that did not show significance in bootstrap cross validation test at $p < 0.01$ are filled in black. Group A and B neurons are shown in magenta and cyan, respectively in (A), (B), (D) and (E). The order of neurons is the same as Fig 2C.

B. Consistency across 15 models in each box in (A) were tested by Wilcoxon's rank-sum test and p values are shown in color codes. Darkness of the color show –log10(p), while red colors show positive mean weights and blue colors show negative mean weights. (A) and (B) show part of the table for sample 1 as examples.

C. Mean (line) and standard deviation (light blue shade) of estimated synaptic weights at each lag for each sample.

D. Estimated synaptic weights for all samples. In this figure, estimated weights from 15 models in each box of (A) are averaged, and shown in 24 x 3 arrangement, as shown in Fig 7C.

E. Consistency across 24 samples in each box in (C) were tested by Wilcoxon's rank-sum test and p values are shown in color codes as in (B). Darkness of the color show –log10(p), while red colors show positive mean weights and blue colors show negative mean weights.

F. Mean (line) and standard deviation (light blue shade) of estimated synaptic weights at each lag for all samples.

File 20 Fig. Mean synaptic weights from each sample are shown as graph representation

Only the weights that showed consistency at FDR < 0.005 among direct synaptic connections are shown.

File 21 Fig. Results of virtual optogenetics experiments

A. Mean response of neurons in the y axis when neurons in the x axis was stimulated.

B. Number of qualified samples for the virtual optogenetic experiments.

File 22 Fig. Number of "presynaptic neurons" used for the prediction models

(A) Number of (directly connected) presynaptic neurons in the connectome data.

(B) Number of directly connected presynaptic neurons observed and annotated in 4D imaging and included in the models.

(C) Number of "indirectly linked" presynaptic neurons. These include directly linked presynaptic neurons and those presynaptic to unobserved or unannotated presynaptic neurons. All target neurons in all samples were included in (A–C).

File 23 Fig. Outline of gKDR-GMM

(A, B) For a given sample j, the model learns to predict activity of the target neuron (neuron i = neuron O in this figure) ahead of time ($y_{i,j}(t+\Delta t)$) from previous activity of presynaptic neurons. Activity of presynaptic neurons was time-delay embedded. k' steps of previous activity in intervals τ' were taken and made a row vercor $X_{m,j}(t)$. These vectors are concatenated for all presynaptic neurons for neuron i ($X_{i,j}(t)$) (= $X(t)$ in this figure). Then gKDR reduces the dimension of presynaptic activities $X_{i,j}(t)$ to K dimensional value $U_{i,j}(t)$ (= $U(t)$). GMM models the joint probability of ($U_{i,j}(t)$, $y_{i,j}(t+\Delta t)$) as a weighted sum of Gaussian distribution which is fitted to the real data. Conditional probability of $y_{i,j}(t+\Delta t)$ is determined from the GMM model and used for prediction.

(C) Toy example explaining gKDR. This example represents an imaginary function $y=f(x_1, x_2, x_3)$. y value is color-coded in the 3D model. If given K=2 (reduction dimension), from the dataset of n samples of (x_1, x_2, x_3, y), gKDR determines the basis vectors (b_1, b_2) because y is independent of b_3 , which defined the rest of the dimensions spanned by x_1, x_2, x_3, while y depends on b_1 and b_2. b_1 and b_2 constitute column vectors of B.

File 24 Fig. Simulation results by gKDR-GP

A. K=3, **B**. K=4, **C**. K=5.
