# Supplementary material for: Ensemble dynamics and information flow deduction from whole-brain imaging data
Source: PLoS Comput Biol. 2024 Mar 15;20(3):e1011848. doi: 10.1371/journal.pcbi.1011848 (PMC10942262; doi:10.1371/journal.pcbi.1011848)
Supplement: Attachment — Submitted filename: Response to Reviewers 20231124.docx [file pcbi.1011848.s002.docx]

**Our Response to Reviewers’ Comments:**

The reviewers’ remarks are underlined and numbered, followed by our one-by-one responses in the following pages.

Please note that all supplemental figures are available from the private link on FigShare ( [https://figshare.com/s/a620af4bc16fff78cd76](https://figshare.com/s/a620af4bc16fff78cd76) ).

Reviewer #1: Unfortunately,I could not assess the paper as most of the figures (even when downloading the tiffs) had unreadable labels.

**Our response:**

We apologize for the inconvenience. In the revised manuscript we provide figures in a vector format, which will not suffer from the low resolution problems.

Reviewer #2: The Toyoshima et al. manuscript uses time-lapse 3D imaging of the whole nematode head and fits a phenomenological model that is constrained by the C. elegans connectome. The authors combined time-delay embedding and independendent compoenent analysis to derive the component dynamics (called "motifs") of the whole brain activities they recoreded from several animals. Then, the authors further constructed time series prediction models of synaptic communications by using dimension reduction and probabilistic modeling. The general idea of using computational modeling to investigate the functional and behavioral role of the connectome is good and timely. However, several points are technically unclear and, partly due to this, some results are weak. The authors need to discuss more openly the limitations of their imaging and their modeling methods and how they pertain to the interpretation of their results.

**Our response:**

We are delighted to see the comment “The general idea of using computational modeling to investigate the functional and behavioral role of the connectome is good and timely”, and we thank the reviewer for providing positive feedback to our study.

Major comments:
1. Most importantly, the authors never actually imaged the whole-brain for a period of time sufficient for their analysis. Line 351ff: “This was expected since approximately half of the neurons were missing from our data because of the difficulty in either observation or annotation (the maximum number of annotated non-random neurons was 110 out of ~190 head neurons).” This even makes the title which mentions “whole-brain” imaging inaccurate. “Whole-head” might be more accurate. Missing neurons cause major problems for a connectomically constrained “whole-brain” analysis and the authors work-around is to add presynaptic targets as indirect links to fill the void. The authors say “[…] this provided better results […]”. This is not surprising, since these indirect links add dynamically unconstrained extra parameters to the model, which will of course make any model fit better. Although I understand the limitations on the experimental methodogy, the authors need to explain clearly why they think they can make claims about “whole-brain” dynamics without imaging the whole brain.

**Our response:**

We appreciate the thoughtful comments by the reviewer. As pointed out, there is a technical difficulty in observing all neurons, which is well recognized in the scientific community. Apart from microscopic issues (resolution, speed etc.), there is a specific issue in annotation. Although all neurons have been identified and named in *C. elegans*, annotating all neurons in an individual animal has not been achieved in any study so far, because positions of neurons are variable between individuals. Even by using NeuroPAL animals, in which neurons are marked by three fluorescent indicators driven by cell-specific promoters, as far as we know the largest number of annotated neurons in a single animal is 155 out of ~190 (Atanas et al., doi: 10.1016/j.cell.2023.07.035). In spite of this, the type of imaging as we perform in the current study is generally called “whole-brain imaging” (for example, Nguyen et al., doi:10.1073/pnas.1507110112; Chaudhary et al., doi: 10.1038/s41467-022-32886-w; Randi and Leifer, doi:10.1016/j.conb.2020.11.001; Wen et al., doi: 10.1016/j.neuron.2020.05.005; Wu et al., doi: 10.1371/journal.pcbi.1010594; Chen et al., doi: 10.1016/j.neuron.2020.05.005 etc.). Please note that *C. elegans* does not have a brain under canonical anatomical definitions, but observing the whole head nervous system, which include major ganglia, is called “whole-brain (calcium) imaging” or “whole-brain recording”.

Because of this difficulty, it is inevitable at the moment that different subsets of neurons are annotated in each animal, and deducing the “whole” dynamics presents a challenge to all researchers in the field. One approach we provide in this study involves TDE-matrix factorization, which reconstructs neural activities of multiple samples that include missing values based on dynamics motifs common across animals. While we recognize there are differences between individuals, matrix factorization approach is designed to extract common dynamics. The fact that we observe the same relationships of motif occurrences between different animals (Revised Fig 5D, E, S5 Fig pages 25-48 and 51-141) verifies that the goal of this approach has been achieved at least in part.
As for gKDR-GMM, including all neurons, rather than (indirectly) connected neurons, for presynaptic explanatory variables causes a poorer reproduction of the real network dynamics. Therefore, in fact, it is not true that the more we include, the better is the result. In this study, we provide two options, “indirect” and “direct”, the latter simply includes only observed and directly connected neurons, which still reproduces real neural dynamics though to a compromised extent, while the former is presented as one possible approach for partly rescuing unobserved neurons, because activity of the unobserved neurons is expected to be related to that of some of its presynaptic partners. We revised the manuscript to clarify these points as follows: " Since this approach (indirect links) provided better results (S10 Fig), we proceeded with the analysis based on the indirect links model, though our codes still support modeling with direct links." (lines 395-397).

2. The authors relegate the methods of the imaging methods almost entirely to reference 23. This is fine to focus the methods section on the analysis/model that is central to the paper. However, the authors should strongly consider including important aspects (those that pertain to the modeling in the manuscript) of the imaging into the present manuscript. For example, there is no mention of the temporal sampling frequency of the 3D imaging, which is critical for any discussion about dynamics. Even reference 23 is not entirely clear. It says there that “The exposure time was 500 ms”. I assume that is the exposure time for the entire cube and not just for a single plain, since the z-plane contains 50 voxels and 50*500ms would be too slow for any claims about dynamics.

**Our response:**

We apologize for the lack of clarity in our explanation regarding the experimental setup. The temporal sampling frequency of the volumetric imaging is about 4 volumes per second, as written in the Method section (line 832-833, lines 678^679 in the original manusrcipt). According to this comment, we added the explanation of sampling frequency also in the Result section, line 103. We also added Fig 1, which illustrates the experimental setup.

3. On the critical role of noise: “Moreover, gKDR-GMM revealed the critical role of noise in realistic neural networks and enabled realistic simulation of whole-brain activities.” In biophysical models the biggest source of noise is from the activity of unknown presynaptic neurons. In a whole-brain model where the activity of every neuron is known, what do the authors believe is the source of the noise and are all noise sources going to be equally critical for network dynamics.

**Our response:**

We appreciate the reviewer’s starting this critical discussion, because this is one of our key claims, and some of the readers are likely to have the same question. First, as the reviewer pointed out, we base our model on observed activities, and predict the activity of the postsynaptic neuron based on those of presynaptic neurons. Therefore, if the noise comes from the (stochastic) activity of a presynaptic neuron, it will be already reflected to the postsynaptic neuron in the model. However, in our model, the relationship between presynaptic and postsynaptic activities are modeled by probabilistic distribution, which is roughly equivalent to the predicted expectation value plus noise. The source of this noise could be noisy synaptic transmission or noisy activity of the postsynaptic neuron, both being possibilities. In our simulation, we added time-independent gaussian noise, which means that the time of occurrence of a deviation from the activity level expected from presynaptic activities is random. This unpredictability of exact time of occurrence is in fact regularly observed in electrophysiological recording of neural activities. Even with the same sensory stimulus, time of occurrence of neural spikes are different in each trial, and only after averaging, the common trend is appreciated (for example, Wang et al. 2023, https://doi.org/10.7554/eLife.90431). Most *C. elegans* neurons do not show action potentials carried by Na^+^ channels but instead calcium spikes are often observed. Because the size of *C. elegans* neurons are small, it has been estimated that only a small number of ion channels are present on a single neuron (Goodman and Lockery 1998, doi: 10.1016/s0896-6273(00)81014-4). If it is the case, time of occurrence of spontaneous or evoked surge of depolarization will be affected by probabilistic opening of each channel, a well-known intrinsic property of ion channels. In fact, time and size of calcium transients are variable even in isolated neurons in culture (Kobayashi et al. 2016, doi: 10.1016/j.celrep.2015.11.064). We assume that this type of neuron-intrinsic stochastic activities are the major source of noise present in the neural network.

For the last question, we previously tried to add noise to only selected neurons, and in fact the effect is different depending on the choice of the neurons. There seem to be a set of neurons, adding noise to which generates the major dynamics observed in real recordings. We decided to leave out the results because there are indefinite numbers of combinations in the choice of neurons to add noise, and evaluation of the results is not straightforward. Because this is an interesting and potentially important issue for understanding the neural network dynamics, the questions will need to be addressed in future studies.

We added considerations on the possible source of noise in Discussion (lines 752-761).

4. The NaCl gradient behavior seems to be happening on a particularly slow timescale and neurons that are not correlated with the behavior are likely excluded from the model because line 283f: “In these models, we only considered annotated neurons with non-random time series activity (time series with considerable autocorrelation).” The authors should discuss whether/how their results relate to the broader behavioral repertoire of C. elegans. To me, it seems that the results only apply to the specific behavior investigated. If that is the case this should be more clearly mentioned.

**Our response:**

We are glad to be given this opportunity of clarifying our experimental constraints and limitations in interpreting the results. As explained in Materials and Methods and Fig 1, which was added in this revision, we performed our imaging experiments in an “olfactory chip” which restrains the animals in a small channel. Therefore, though they are stimulated by changes in salt concentrations as a sensory stimulus at the nose tip, the animal cannot move more than just wiggling motions. Therefore, the experiment is not intended to observe behavioral outputs, though wiggling movement can sometimes reflect the intention of the animal to move forward or backward. As described in the manuscript, forward-backward intentions can be also read out by looking at the forward-backward command neurons (group A and B in the text). The behavioral strategy called klinokinesis is based on a slow salt concentration change which affects the forward-backward switches. This is why we stimulated the worms in a slow time course. On the other hand, there are also faster sensation and responses. A typical behavioral example is klinotaxis, which is based on a faster salt concentration change at the frequency of head swing, whose period is a few seconds. Also, Kato et al. (doi: 10.1016/j.neuron.2013.11.020, 2014) applied random changes of sensory stimulus (with M sequence) and found both faster and slower components in the response of sensory neurons. Our current setup is likely missing these types of fast response in the neural circuit. Also, the head swinging movement has been reported to depend on proprioceptive feedback from body bends to head motor neurons. Again, this type of response is missed because the worms cannot perform head swing in the olfactory chip.

We added statements on the limitations of our setup and relevance of our observations to klinokinesis in Discussion (lines 768-781).

5. Line 59f: “Therefore, the network’s shape and synaptic properties are the primary determinants of information flow.” I somewhat disagree with this, since I would also consider intrinsic properties (and their changes at neuromodulator time-scales) primary determinants of information flow but I guess it depends what one considers “primary”. However, if the authors believe that their results pertain to the importance or irrelevance of intrinsic properties, I’d be interested to find a broader discussion of connectomics vs intrinsics in the manuscript.

**Our response:**

We thank the reviewer for pointing out the shortage of our description. We actually do not intend to assert that intrinsic properties are not involved. As described in main text and Materials and methods (also Fig 6A and S23B Fig in revised manuscript), our model predicts the activity of the postsynaptic neuron based not only on presynaptic neurons but also the own previous activity of the postsynaptic neuron (depicted “target neurons” in the figures). It means that the dynamic property of the neuron itself (periodic, bistable etc.) is in principle learned by the model, and it is an important determinant of the dynamic behavior of the network. The important role of the prior activity of the neuron itself may be also appreciated in the simulation shown in newly added Fig 10B, control trials.

To avoid misunderstandings, we changed line 59f (line 60-61 in the revised manuscript) to “Therefore, in addition to the intrinsic properties of constituting neurons, the network’s shape and synaptic properties are the primary determinants of information flow.”

6. In the time-delay embedding/ICA, I wonder whether and how the motifs of neuronal dynamics depend on the choice of a time window for these analyses. For example, some motifs (e.g., motifs 1 and 2) look like mirror images of half-cycles of an oscillatory activity pattern, but the detection of these motifs will depend on the choice of the window size. For instance, why does the method extract a complete oscillation cycle as a motif? Is the optimal window size for time-delay embedding autoatically determined by the method or chosen arbitrally by experimenters?

**Our response:**

We thank the reviewer for this important point. As the reviewer pointed out, the length of the time-window (or the number of time-delay steps) affects the results to some extent. According to this comment, we provided two additional analyses for the effect of the number of time-delay steps.

By using simple time series, we confirmed that the motifs are generally oscillatory waves with phase shift (see S6 Note). We also found that the number of time-delay steps affects the cycle of oscillatory waves covered by the motif.

When the number of time-delay steps is smaller than the typical frequency of the original time series, the motifs form a part of a cycle of oscillatory waves. The spontaneous and synchronized activity observed in our whole-brain data seem to have a larger typical frequency compared to the time-delay steps. This will be the case for the motifs 1 and 2.

When the number of time-delay steps is larger than the typical frequency of the original time series, the motifs cover multiple cycles of oscillatory waves. The typical frequency of stimulus and corresponding responses is smaller than the time-delay steps. This will be the case for the motifs 13 and 14.

These phenomena may be explained as follows. Each time series separated by time-delay embedding forms oscillatory waves of different phase; motifs took the form of oscillatory waves of different phases so that the separated time series could be expressed as a weighted sum of the motifs. In addition, if the original time series oscillates aperiodically, the separated time series may have a nearly constant value (DC component). In order to approximate such time series, the motifs would be required to be almost complementary. In accordance with these requirements, the motifs will form a pattern that resembles the mirror images of half-cycles of an oscillatory activity pattern. This is the case for the motifs 1 and 2.

However, these findings are based on simple time series and do not always hold true for more complex data including the whole-brain neural activity, which contains multiple typical frequencies. We therefore applied TDE-RICA to the whole-brain neural activity dataset with different time-delay steps (S7 Data). We found that the obtained motifs and occurrences are somewhat consistent among several different numbers of time-delay steps.

We have already tried to find the optimal number of time-delay steps by several methods. An automatic estimation method based on predictability has been reported previously (Ahamed et al. Nature Physics, 2021, doi: 10.1038/s41567-020-01036-8). We have already applied this method to our whole-brain data and estimated the number of time-delay steps was to be about 10. We cannot use this value because when the number of time-delay steps was set to 10, the motifs changed little in time, and the time-delay embedding had little effect. Our whole-brain activity data is noisy and stochastic and might not be suitable for this estimation method. We also calculated the autoregression of neural activities, but the results varied between neurons.

Therefore, it is not possible to find the optimal value for the number of time-delay steps with these methods. Finally, we tested several candidate values and chose 300 as the number of time-delay steps. With this value, the motifs and corresponding occurrences seem to capture the large-scale dynamics of neural activities.

According to this comment, we added a paragraph that refers to the effect of the number of time-delay steps to the Result section (lines 234-243).

7. Line 268ff: "[...] This result suggests individual differences in the quantitative relationships of neurons between animals, despite the general assumption of little individual differences in neural activity in C elegans [...]" Are the individual differences also seen in animal behaviors? The statement does not make much sense to me without knowing the individual differences in neuronal activity and those in animal behavior. The former may be just a reflection of the latter.

**Our response:**

As mentioned above, we performed all experiments reported in this manuscript in olfactory chips, and therefore did not observe the behaviors. In terms of individual difference of behaviors, the question has been nicely addressed by Stern et al., 2007 (doi: 10.1016/j.cell.2017.10.041) and Ji et al., 2021 (doi: 10.7554/eLife.62889). They looked at “roaming” and “dwelling” behaviors and in fact found there is long-term individual variability in the behavior. Our observation of individual variability in neural activities is likely related with this type of behavioral variability. Neural variability may be causing behavioral variability, or vice versa because there are proprioceptive inputs to neurons as discussed above. Stern et al. found that neuromodulation by serotonin affects consistency of behavioral variability, likely through its action on the nervous system.

We added discussion on this. We have already discussed the individual variability of connectomes found in electron microscopy reconstructions reported in 2021 (Witvliet et al., doi: 10.1038/s41586-021-03778-8).

8. Line 335ff: The gKDR-GMM model has two indices i and j, where i refers to neurons observed in samlpe j. This raises a doubt whether the gKDR-GMM model merely analyzes neuronal activty data from different samples (animals) in paralell. How does the method extract features commonly found across different samples? The current manuscript just describes the technical details of the analysis method, but the authors should also explain the conceptual ideas behind it in more detail. In addition, no quamtitative evaluation of the model's performance seems to be attempted in the manuacript, although I cannot deny the possiblity that I missed such information.

**Our response:**

As nicely summarized by the reviewer and as we described in Results, “Unlike TDE-RICA, each sample was treated separately but not combined”. In gKDR-GMM, each sample (animal) was treated separately to generate independent models. A major reason for this is that, as described earlier, different subsets of neurons were observed in each sample. While TDE-RICA could integrate the results from different samples through matrix factorization, there is no good means for combining gKDR-GMM models. We therefore extracted scalar values, gradients of synaptic input-output function, which were depicted as "synaptic weight" in the manuscript, that characterize communication strength and sign between each presynaptic neuron and postsynaptic neuron. We compared these values between samples and selected neuron pairs whose connection strength are consistent across samples. We believe these analyses, shown in Fig 9C-G in revised manuscript, extract important synapses and their contribution to network dynamics that are common across individuals. We also used these measures of synaptic strength for comparison between functional connectivity and structural connectivity by chemical or electrical synapses. Biological insights obtained are described in Results, line 570-581 and Discussion, lines 782-787.

As a part of quantitative evaluation, in the revised manuscript, we added a scatter plot of cross-correlation between all pairs of neurons in real recordings vs those obtained by simulations by the gKDR-GMM model (Fig 7A and S12 Fig in the revised manuscript). Correlation coefficients between these values, which range from 0.232 to 0.919 depending on samples, are summarized in Fig 7B. We hope these plots will allow more visual and quantitative evaluation of the performance of the models. For hyperparameter search, we used mean squared error of the neuron pair cross-correlations for evaluation of the performance under each parameter set (S10 Fig and Materials and Methods). In addition, to evaluate dynamic relationships between neurons, we compared lagged cross-correlation between real data and simulated data and statistical significance is shown on the top of each figure (Fig 7C and S13 Fig). Dynamics of neural ensembles were also compared by TDE-RICA decomposition and comparison of motif occurrences (S15 Fig).

Minor comments:
9. Lines 637ff: “Therefore, there is a pressing need for new methods that offer truly quantitative understanding of the ensemble behaviors.” I find the wording here weird. In what sense are cross correlation and Granger casuality not “truly quantitative”? What does it mean to be truly quantitative? From the authors' mention of Granger Causality, I believe they are here expressing their desire for more causal inference methods in neuroscience as opposed to correlational (xcorr & Granger). If that is the case the authors should feel free to say so. If not, they should explain what makes a method “truly quantitative”.

**Our response:**

In this part of discussion, we intended to convey our vision: “However, these methods are merely descriptive of the relationships between neurons or brain areas, and their results cannot be utilized for long-term prediction or simulation.” (The sentence preceding the cited sentence), and our point is of course not whether quantitative or not. We thank the reviewer for pointing out this self-contradiction. We changed the sentence to: “Therefore, there is a pressing need for new methods that incorporate causal structures of the data and offer predictive capacity.” (lines 791-793).

10. It would be nicer if the distinction between group A and group B were more clearly visible in Fig. 8A-D. For instance, you may use colors for labeling the two groups.

**Our response:**

We thank the valuable comment by the reviewer. As suggested, we labeled group A and group B neurons in magenta and cyan, respectively, in Fig 9A-D and S19A, B, D, and E Fig. Also, the order of the neurons, which were alphabetical in our original manuscript, was changed to that of Fig 2C, which is determined by hierarchical clustering based on cross-correlation across samples and nicely clusters group A and B neurons. The figures look more interpretable than our original figures.

Reviewer #3: Toyoshima et. al. present a powerful analysis of brain-wide calcium imaging data in mechanically restrained C. elegans. Authors have collected a unique dataset comprising annotated neural recordings across 24 animals. Their analysis pipeline is equally unique and commendable in my opinion for being non-parametric and comprehensive in scope. In the same study they have presented interpretable dynamic motifs using a thoughtful combination of delay embedding with reconstruction ICA and modeled the activity dynamics using a probabilistic kernel method to extract a functional connectome as well as neural transfer functions. It is also commendable that the authors have made their data available online. I also appreciated their systematic handling of the hyperparameters to separate the robust vs variable parts of their results.

After some modifications to the writing and overall presentation of their work (described below), I recommend this work for publication and expect it to become an exemplar of neural data analysis in large neural recordings.
I have following suggestions, most of which are related to the presentation of the work and putting it in context with existing literature.

**Our response:**

We are delighted to see the comment that “expect it to become an exemplar of neural data analysis in large neural recordings”, and we thank the reviewer for providing positive feedback to our study.

1. I had a hard time reading the figures because of their low resolution. In Fig. 1 and 8, I couldn't read the neuron names. I also had a hard time seeing the top row in Fig 1., which has the stimulation protocol. These issues stand for all figures, but I felt was particularly important in Fig 1. and 8. Vectorizing the graphics and text would be preferable.

**Our response:**

We apologize for this severe problem. Because the first recommendation at the submission site was tiff files, we chose this format, but at the same time because of size limitation of the submission site we could not make the files in higher resolution. We now realize that this was not a good choice, and as suggested by the reviewer, revised figures are submitted in pdf files in vector formats. We hope the figures are seen more clearly this time.

2. A schematic of their experimental setup along with the microfluidic chip would be good in Fig 1. I was under the impression that the worms were immobilized in the chip, but on line 241-242, authors say “This suggests that when worms move forward in the microfluidic device, their heads inevitably experience mechanical stimulation from the device’s wall.”

**Our response:**

As pointed out by the reviewer, we observed neuronal activities of a worm immobilized in a microfluidic chip, but there is a small space between the wall and worm body, which allows the worm to press a part of the body against the wall when they try to move. To help readers to understand our experiments better, we included a graphical presentation of our experimental set-up in revised Fig 1.

To the same point, some discussions about how much different animals were able to move in the microfluidic device would be appreciated. How much of the neural activity variance could be explained by the movement of the animals within the chip?

**Our response:**

We apologize for the lack of clarity in our explanation regarding the experimental setup. According to this comment, we added Fig 1 that illustrates the experimental setup. As the reviewer pointed out, the worms were almost completely immobilized in the device, but they can move their heads slightly. Sometimes there is a gap between the worm's body and the walls of the device, and the worm sometimes touches the walls when it moves. Although our microscope cannot observe the worms’ body touching the walls of the device, we calculated movement of the worm based on the parallel displacement of the recorded images. We visualized the movement along with the neural activity (S5 Fig). Typically, the worm moves about 20 micrometers back and forth during recording. This movement is slight and appears to have little relationship to the neural activity.

3. In my experience, the time-delay parameter has a big effect on the outcome. In this study, authors use a time-delay equivalent to the NaCl stimulation period (~60s). Which appears long to me, with this window, only the dynamics that are occurring at a minute long timescale are robustly identified, while faster dynamics are smoothed over. Usually, if there is a strong cycle in the data, a delay of about a quarter to a half of the cycle period is used (rule of thumb). I would have expected a delay parameter of the order of 30s to be used. There are systematic ways of optimizing the delay parameter through heuristics such as the autocorrelation timescale, predictability time (Ahamed et al. Nature Physics, 2021), predictive information (Costa et al. Chaos, 2023). It could be revealing to use these methods for optimizing the time delay parameter. If that's not possible, a demonstration of the robustness of motifs with different delay parameters could also be good.

**Our response:**

We thank the reviewer for this important point. According to this comment, we provided several analyses to observe the effect of the number of time-delay steps.

By using simple time series, we tested the effect of the time-delay steps on the motifs and their occurrences (see S6 Note). In the case of periodic oscillatory waves, robust motif occurrences can be obtained if the number of time-delay embedding steps is more than half of the oscillation period. This is consistent with the “rule-of-thumb” mentioned by the reviewer. However, for non-periodic oscillatory waves, the number of time-delay embedding steps may need to be larger than half of the average oscillation period to obtain smooth motif occurrences.

We have already applied the “predictability time” method (Ahamed et al. Nature Physics, 2021) to our whole-brain data. This method estimated the number of time-delay steps to be about 10. We cannot use this value because when the number of time-delay steps was set to 10, the motifs changed little in time, and the time-delay embedding had little effect. Our whole-brain activity data is noisy and stochastic and might not be suitable for this method. We also calculated the autoregression of neural activities, but the results varied between neurons. Therefore, it is not possible to find the optimal value for the number of time-delay steps with these methods. Finally, we tested several candidate values and chose 300 as the number of time-delay steps. With this value, the motifs and corresponding occurrences seem to capture the large-scale dynamics of neural activities.

We added the results of applying TDE-RICA with different time-delay steps to the whole-brain activity dataset as S7 Data. We found that the obtained motifs and occurrences are somewhat consistent among several different numbers of time-delay steps. When we set the time-delay steps as 150 (corresponding to about 30s), motifs corresponding to motifs #13 and #14 in the original results were similarly obtained. Motifs corresponding to motifs #1 and #2 in the original results were also obtained but one of them lost its temporal variation.

According to this comment, we added a paragraph that refers to the effect of the number of time-delay steps to the Result section (lines 234-243).

4. In the result section on the motifs (lines 220-221): “Other neurons, including downstream interneurons, had lower weights, suggesting that activities of sensory neurons might only weakly influence the downstream neurons”. This is a potentially important point, and it would be nice if the authors can verify this. Not only will that add to the biology, but it will also give confidence that the motifs are interpretable. If the above is true, then the future activity of the downstream neurons cannot be predicted well by sensory neurons because it is only weakly influenced by them. Is it possible to test this point?

**Our response:**

According to this comment, we visualized the changes in neural activity induced by NaCl stimulation (Fig S8). Sensory neurons including ASEL/R and BAGL/R showed significant response to the stimuli on average. Interneurons and motor neurons also responded to the stimuli in some cases, but in others they did not respond or responded in the opposite direction, showing large individual differences. Such variability diminishes the average response of the neurons. Even in neurons that responded on average, the magnitude of the response was small compared to the overall variance. These trends on average are consistent with the results of TDE-RICA, suggesting that this analysis successfully extracted common behaviors among individuals as motifs. In addition, considering that several neurons involved in forward and backward movement showed clear spontaneous and synchronous activity, it would be difficult to predict the activity of these neurons based solely on sensory input.

We added a paragraph that refers to this additional analysis to the Result section (lines 263-273). Also, Fig 7E and S16 Fig (originally Fig 6C and S12 Fig), which are presented also for evaluation of sensory-related periodicity of neuronal activities, were revised to make them more interpretable.

5. The statement on lines 397-398: “Upon surveying all results, it appears that there must be distinct groups of correlated neurons for the dynamics to be accurately reproduced”, needs be clarified a little. I would’ve expected that when the neurons exhibit globally correlated activity, their activity would be easier to reproduce. But it seems the opposite is true.

**Our response:**

Although theoretical analysis or modeling of network dynamics is out of scope of this study, we speculate that epilepsy-like dynamics such as those seen in sample 6, 19 and 20 in Fig S11B is difficult to reproduce because our model is based on synaptic connectivity. If multi-step synaptic interactions are involved in the model, it necessarily causes a delay, or a sequence of activation, and therefore if a vast majority of neurons are simultaneously activated and multiple multi-step interactions are required because of the network structure, it will cause limitations in reproducing such dynamics by the model. In addition, even if simultaneous activation is achieved by the model, stability of this state may not be the same as that of the real network, such as being too unstable, as seen in the simulation result of sample 19 in Fig S11B. We suspect that the stochastic epilepsy-like dynamics observed in our imaging experiments may be carried by extra-synaptic secreted signals such as monoamine neurotransmitters or neuropeptides (for example by serotonin, Fig. 6 in Dag et al. https://doi.org/10.1016/j.cell.2023.04.023).

We added the following sentence in Discussion (lines 450-452) to propose this possibility: "We speculate that such simultaneous activation of a vast majority of neurons may be caused by extrasynaptic signaling, such as those by monoamine neurotransmitters or neuropeptides as observed recently[16]"

6. I like the analysis that the authors do to identify functional connections and time lags using their predictive model. It would be nice if the authors can compare their results (both the connections and lags/decay of responses) to the recent preprint by Randi et al. (bioRxiv 2023) from Andy Leifer’s lab, where an estimate of the functional connectome was presented by direct stimulation of neurons. How close does an estimate of based on predictive modeling of the dynamics get to an experimentally derived estimate?

**Our response:**

We chose to perform a more direct comparison of our gKDR-GMM model and Randi et al. Because our gKDR-GMM is a generative model, we can perform simulation with artificial manipulation that mimics optogenetics. The results are described in a new section “Virtual optogenetics to assess propagation of neural activities through the network” in Result, Fig 10 and S21 Fig. We note that the consequence of virtual optogenetic stimulation is highly variable and the major reason for it is the difference in network states at the time of stimulation, as appreciated in our simulation in Fig 10B. This was discussed by Randi et al, but was not directly tested, because it is impossible to fully control network state and perform control experiments (no optogenetic stimulation) at the same time in real animals. Modeling works have the opportunity to perform these controls, which revealed the large influence of network states, and we assessed the effect of optogenetic stimulation by directly subtracting the results of control experiments. This provided the neuron to neuron signal propagation map shown in S21A Fig. The results also suffer from animal to animal variability. Fig 10C provides direct comparison between our results with those by Randi et al. As appreciated from these figures, if sufficient number of experiments were performed, the neuron pairs show considerable positive correlation between our results and Randi et al., underscoring the validity of our models. We added description of this analysis as a new section "Virtual optogenetics to assess propagation of neural activities through the network" in Results (lines 621-660). We thank the reviewer for this creative advice.

7. The discussion is very thorough in its handling of the computational methods and various details, but I found the discussion lacking in its handling of the biology of the system. What are the key biological insights that were gained from the analysis? How reliable are they? What do they suggest about the system? Do they suggest any follow-up experiments? What are the main biological takeaways? These points are there in the paper (even in the abstract), but it would be nice to consolidate and expand upon them in the discussion.

**Our response:**

We appreciate the reviewer for pointing out the unbalance and encouragement to add discussions on biological insight obtained from our analyses. As described in our response to the questions by reviewer #1, one of our motivations was to understand underlying neural circuit mechanisms for behavioral responses to sensory stimuli. Especially, we have previously been working on the mechanisms of salt chemotaxis. Two major behavioral mechanisms have been found, klinokinesis and klinotaxis, by others and us (Pierce-Shimomura et al, 1999, doi: 10.1523/JNEUROSCI.19-21-09557.1999; Iino and Yoshida, 2009, doi: 10.1523/JNEUROSCI.3633-08.2009; Luo et al. 2014, doi: 10.1016/j.neuron.2014.05.010). Of these, klinokinesis is based on a probabilistic response to salt concentration changes in a relatively slow time scale. We have found behavioral correlates of interneuron activities, but because killing a single class of interneuron does not abolish chemotaxis, existence of multiple pathways have been suggested (Sato et al, 2021, doi: 10.1016/j.celrep.2021.109177; Mabardi et al, 2023, doi: 10.1016/j.neures.2022.10.004). Upon revision of the current manuscript, we focused on how sensory information is transmitted. In S8 Fig, we show that salt sensory information is in fact transmitted to the interneuron circuit. On the other hand, as described in Results, there are prominent spontaneous activities in command interneurons, thus the effect of sensory inputs is relatively weak. Another important finding is that the response of interneurons is variable between individuals. Synaptic weight estimation by gKDR-GMM also indicated that the interactions within command neuron circuit is invariable, while sensory neurons affect the interneuron circuit in multiple pathways which are variable between individuals (Fig 10A). Therefore, we propose an overview that the sensory-motor regulation is based on a robust but stochastic core motor circuit, and its regulation by weak, multi-threaded and variable sensory transmission pathways. This discussion was added to Discussion (lines 762-787) and briefly to Abstract and Introduction.

Our work also strongly suggested the importance of noise in driving the network dynamics. Upon revision, we stressed this point again in Discussion. At the same time, we discussed that the noise is likely an intrinsic property of *C. elegans* neurons. Therefore, spontaneous and coordinated activities of *C. elegans* nervous system is driven by strong synaptic contacts and intrinsic noise of the component neurons. We expect that this discussion (lines 752-761) is also helpful for conveying biological insights to readers.
